# Socio-economic predictors of uptake of malaria interventions among pregnant women and mothers of under 5 children in Oyo State, Nigeria: a cross-sectional study

**DOI:** 10.11604/pamj.2023.44.65.27885

**Published:** 2023-02-02

**Authors:** Kelechi Elizabeth Oladimeji, Joyce Mahlako Tsoka-Gwegweni, Felix Emeka Anyiam, Sanni Yaya, Jerry John Nutor, Gbenga Abiodun, Claude Ngwayu Nkfusai, Doaa Hegazy, Oluwafunmilola Deborah Awe, Daniel Ter Goon

**Affiliations:** 1 Department of Public Health and Laboratory Medicine, Faculty of Health Sciences, Walter Sisulu University, Eastern Cape, South Africa,; 2 Faculty of Health Sciences, University of the Free State, Bloemfontein, South Africa,; 3 Center for Health and Development (CHD), University of Port Harcourt, Port Harcourt, Nigeria,; 4 School of International Development and Global Studies, University of Ottawa, Ottawa, Canada,; 5 The George Institute for Global Health, The University of Oxford, Oxford, United Kingdom,; 6 Department of Family Health Care Nursing, School of Nursing, University of California, San Francisco, San Francisco CA, USA,; 7 Department of Mathematics, Southern Methodist University, Dallas, Texas, USA,; 8 Cameroon Baptist Convention Health Services (CBCHS), Yaounde, Cameroon,; 9 The Egyptian Ministry of Health and Population, Cairo, Egypt,; 10 Africa Centre for Enhanced Policy and Development, Nigeria,; 11 Department of Public Health, University of Fort Hare, Eastern Cape, South Africa

**Keywords:** Socio-economic status, pregnant, women, malaria intervention, Ibadan

## Abstract

**Introduction:**

socio-economic status (SES), especially for women, influence access to care. This study aimed to determine the relationship between SES and uptake of malaria intervention by pregnant women and non-pregnant mothers of children under 5 years old in Ibadan, Oyo state, Nigeria.

**Methods:**

this cross-sectional study was conducted at Adeoyo teaching hospital located in Ibadan, Nigeria. The hospital-based study population included consenting mothers. Data were collected using an interviewer-administered modified validated demographic health survey questionnaire. The statistical analysis involved both descriptive (mean, count, frequency) and inferential statistics (Chi-square, logistic regression). Level of statistical significance was set at 0.05.

**Results:**

mean age of the study´s total of 1373 respondents was 29 years (SD: 5.2). Of these, 60% (818) were pregnant. The non-pregnant mothers of children under five years old showed a significantly increased odds (OR: 7.55, 95% CI: 3.81, 14.93) for the uptake of malaria intervention. Within the low SES category, women aged 35 years and above were significantly less likely to utilize malaria intervention (OR=0.08; 95% CI: 0.01-0.46; p=0.005) compared to those younger. In the middle SES, women who have one or two children were 3.51 times more likely than women with three or more children to utilize malaria intervention (OR=3.51; 95% CI: 1.67-7.37; p=0.001).

**Conclusion:**

the findings provide evidence that age, maternal grouping, and parity within the SES category can significantly impact on uptake of malaria interventions. There is a need for strategies to boost the SES of women because they play significant roles in the wellbeing of members of the home.

## Introduction

In Africa, children under five years old and pregnant women are worst hit by the devastating burden of malaria infection despite available effective malaria preventive interventions such as insecticides treated nets (ITNs) and antimalaria medicines [1]. Nigeria, a middle-income country in sub-Saharan Africa accounts for the highest burden of malaria in Africa and globally [1,2]. As part of strategy to curb the malaria epidemic, the United Nations (UN) General Assembly in the year 2015 adopted the agenda of the Sustainable Development Goals (SDG) in which goal 3 was directed at reducing 90% of malaria burden and maternal and neonatal deaths by the year 2030 [3]. Unfortunately, global and national progress made in significantly reducing malaria morbidity and mortality in endemic regions since the year 2015 has been stalled by gaps in access to core malaria interventions [4]. Compelling evidence reveals that socio-economic status (SES) which is defined as a composite measure of an individual´s economic and sociological standing based on income, education, and occupation, is an important determinant of health [5] which impact on the malaria burden calls for more research. Based on non-empirical evidence, it is widely accepted as a principle that wealthy people tend to be in better health than people of poorer status [5]. This attests to the statement by World Health Organization (WHO) that “multidimensional poverty is a determinant of health risks, health-seeking behavior, health care access and health outcomes” [6]. Further, a considerable number of studies have highlighted that SES significantly influence the effective management of malaria and treatment outcomes among households in Nigeria [7-9]. A clear understanding of the impacts of SES such as housing structure, education, occupation, income, and wealth on uptake of malaria interventions can help to better design socio-economic interventions to control and eliminate the disease [10]. However, few or no studies in the country have examined SES as predictors for uptake of current malaria interventions especially by pregnant women and mothers of under 5 age children. Literature based evidence from economic and social sciences has indicated that women´s SES plays a catalytic role in ensuring improved health outcomes of a child and the wellbeing of the entire household [11]. To investigate the socio-economic factors that promote malaria intervention uptake among pregnant women and mothers of under-five aged children in Ibadan, Oyo State, Nigeria.

## Methods

**Study design and setting of the study:** this study is part of a cross-sectional study carried out on consenting women who came for clinic visits at the study site between May and November 2016. The study location was Ibadan, a city located in Oyo State and situated in the south-west of Nigeria. Ibadan city has 11 local governments area (LGAs); five LGAs within the metropolis and six LGAs at the periphery of the metropolis. The LGAs include Egbeda, Oluyole, Akinyele, Ona-ara, Lagelu, Ido, Ibadan North East, Ibadan North West, Ibadan South East, Ibadan South West and Ibadan North. The total population of Ibadan was about 2.5 million from the last national census conducted in 2006 and is currently estimated to be over 3 million [12]. While the majority of Ibadan residents are traders, many are civil servants and farmers producing a variety of agricultural products that contribute to the food system serving the urban population [13]. The climatic condition characterized by warm temperature, humid conditions, and high rainfalls in Nigeria and in the study location makes the setting endemic for malaria with detrimental impact on pregnant women and children aged under five years old [14]. Adeoyo Maternity Teaching Hospital (AMTH) situated in Ibadan was used as the study site for consenting participant enrollment and data collection. This hospital is a 135-bed tertiary healthcare facility specialized in offering obstetric and gynecologic services with over 4500 deliveries per year. It also serves as a referral center for primary and secondary healthcare as well as private hospitals in Ibadan and its environs [15].

**Study population and sampling:** the study population consisted of pregnant women and mothers of under 5 children randomly selected and enrolled in the study after consent was obtained. A multi-stage sampling technique was applied in the study, and it involved; firstly, the identification of Ibadan as study location, secondly AMTH as the study site and lastly, random recruitment of consenting study participants into the study. The pregnant women were recruited from the antenatal clinics (ANC) in the study site while the mothers of under 5 children were recruited from the study site´s children outpatient clinic.

**Sample size estimation:** the Cochrane formula [16] for cross-sectional studies were used to calculate the population size. The sample size N (581) for pregnant women was estimated using a prevalence (p) of 40% [17]. Similarly, estimated sample size for mother´s of under 5 age children to be recruited into the study was 476 using the prevalence rate of 54.1% [18]. In both calculations, an assumed attrition rate of 20% was considered.

**Context of study (justification for study maternal grouping, overview of malaria intervention and accessibility in the study setting):** in this study, the maternal category - pregnant women and others of children aged under five years were targeted as the study population based on evidence which has shown that children under age 5 and pregnant women are the groups most vulnerable to the illness and death from malaria infection in Nigeria [1]. Besides, children under the age of five are too young to take part in this study, which is why their mothers were used. Also, previous reports have shown that mothers are primary carers of children in this age group and will ensure their children's well-being by seeking quality healthcare [19]. Accordingly, malaria preventive intervention uptake by mothers of children aged under five years and pregnant women that this study investigated refers to malaria preventive measures outlined by the WHO and Nigerian malaria guidelines. Women complying with either one of the malaria preventive intervention mentioned were said to have an uptake. These measures include: a)Intermittent preventive treatment of malaria during pregnancy (IPTp) through free administration of at least 3 doses of sulfadoxine- pyrimethamine (SP) medication to all pregnant women during their ANC visits; b) use of insecticide treat nets (ITNs) which was made available to all health facilities in the country to freely distribute to everyone, thanks to sponsorship from international donors like WHO, United States Agency for International Development (USAID) and United Nation (UN). However, for several reasons, one of which is high demand since it is most widely practiced malaria preventive measure, free access to these ITNs are affected. This has led to the buying of these nets from other outlets like privately owned pharmacies since 2015; c) seasonal malaria chemoprevention which involves the intermittent administration of complete doses of antimalarial medicine sulfadoxine- pyrimethamine plus amodiaquine (SP+AQ) during the malaria season to prevent malarial illness by maintaining therapeutic antimalarial medicine concentrations in the blood throughout the period of greatest malarial risk. This chemoprophylaxis is similar to IPTp-SP given to pregnant women but the target group for SP-AQ is children aged under five years and only the health facility provides access to this medication.

**Data collection:** to achieve our study objective, we adapted and modified demographic and SES questions from the demographic health survey (DHS) questionnaires [20]. The data collection tool (semi-structured questionnaire) was interviewer administered to consenting participants. The SES section of the questionnaire assessed ownership of household assets, and also questions on monthly household income in local currency. The questionnaire was developed in English and translated into the predominant local language, Yoruba language and back-translated to English language for quality assurance.

**Measures and variables´ computation:** in classifying the SES, we followed definitions from the Kuppuswamy´s socio-economic status scale, which classified the study populations into high, middle, and low [21]. Based on the modified household asset indicators, [20] the composite relative and household asset variables used for the SES computation included television (No, yes, if yes, how many), refrigerator (No, yes if yes, how many), Vehicle (No, yes, how many), motorbike (No, yes, if yes, how many), home telephone (No, yes), mobile telephone (No, yes, if yes, how many) washing machine (No, yes), microwave (No, yes, if yes how many), indoor bathroom (No, yes, if yes, how many) and computer - laptop/desktop (No, if yes how many).

**Outcome variable:** the main outcome variable was uptake of malaria intervention (Yes or No).

**Explanatory variables:** included sociodemographic factors such as maternal grouping (Pregnant women and non-pregnant mothers of under 5 children]), age groups (24 year and/or less, 25 to 34 years, 35 years and older), marital status (never married, married, separated /widowed), educational level (no formal education, primary, secondary, tertiary ), religious (Christian, Islam and traditional worshipers), status of residence (owned, not owned and others), composite SES measure (1=high SES and 0=low SES), parity (no child yet, < 3 children and ≥ 3 children) and various malaria preventive methods.

**Statistical analysis:** data analysis was conducted using the Statistical Package for Social Sciences (SPSS) v25 after it had been imported, coded and cleaned from the data extraction sheet. Categorical data were presented in the form of frequencies and percentages (%) and summary statistics in means and standard deviations (SD) with results presented in tables. Bivariate analysis was performed to determine the risk association (using odds ratio, ORs). All ORs were reported with their 95% CI and corresponding p values. Multivariate analysis was also done to adjust for the effect of confounders. Predictor variables that were statistically significant during the bivariate logistic regression were put into the multiple logistic regression model to determine cofounders, and in order to examine which significant predictor variable best predict a certain outcome as a significant independent risk factor. An observation was said to be statistically significant if the “p-value is less than or equal to 0.05 (≤0.05) at a 95% confidence interval.

**Ethical considerations:** approvals to conduct the study were obtained from the Oyo state ministry of health ethics committee (AD13/479/1035) in Nigeria and the biomedical research ethics committee (BREC- BE199/16), University of KwaZulu-Natal, South Africa. In line with the core ethical principles, signed informed consent was obtained from the consenting study participants and they were assured of the confidentiality of their information especially during dissemination of study findings.

## Results

**Socio-demographic characteristics of the study population:** a total of 1373 women were recruited for the study, with over a half, 59.58% (818) pregnant and the rest, 40.42% (555) non-pregnant mothers of children underage 5 years. The ages of the respondents ranged from 19 to 47 years, with a mean (SD) age of 29.27 (5.20) years. Most of the respondents, 76.84% (1052) at the time of the study were at the middle SES, were married, 91.84% (1261), had at least a secondary level of education, 51.64% (709) and were Islam in their religious affiliation, 57.10% (784). Most of the respondents, 73.63% (1011) said they do not own their place of residence while 23.82% (327) said they do. About a third, 20.03% (275) of all the respondents had no child, while over a half, 52.73% (724) have had two children or less and 27.24% (374) have had three (3) or more children as shown in Table 1.

**Table 1:** distribution of socio-demographic characteristics (N=1373)

Variables	N	%
**Maternal grouping**		
Pregnant women	818	59.58
Non pregnant mothers of under-5	555	40.42
**Age group (years)**		
<24	207	15.08
25-34	954	69.48
35 - 47	212	15.44
Mean ± SD	29.27±5.20	
**SES (n=1369)**		
Low SES	202	14.76
Middle SES	1052	76.84
High SES	115	8.40
**Marital status**		
Never married	42	3.06
Married	1261	91.84
Separated/widowed	70	5.10
**Education**		
No formal education	97	7.06
Primary	81	5.90
Secondary	709	51.64
Tertiary	486	35.40
**Religion**		
Christianity	567	41.30
Islam	784	57.10
Traditional worshiper	22	1.60
**Status of residence**		
Owned	327	23.82
Not owned	1011	73.63
Others	35	2.55
**Parity**		
No child	275	20.03
< 3 children	724	52.73
^3^3 Children	374	27.24

### Uptake of malaria intervention

**Socio-economic predictor for the uptake of malaria intervention:** most of the respondents, 92.12% (830) reported to have used at least one of the malaria intervention while a small proportion of 7.88% (71) used none of the intervention (Table 2). The study assessed the socio-economic predictor for the uptake of malaria intervention by mothers using bivariate analysis. From the bivariate logistics regression model (Annex 1), no significant associations for the uptake of malaria intervention were found for low, middle and high SES (OR: 1.57, 95% CI: 0.38, 6.47, p=0.533; OR: 2.55, 95% CI: 0.78, 8.32, p=0.120).

**Table 2:** socio-demographic and clinical predictors of uptake of malaria intervention using multivariate logistic regression (Adjusted OR)

Variables	Malaria intervention		OR (95% CI)	p-value
	Yes (n=830) Freq (%)	No (n=71) Freq (%)		
	[95%CI]	[95%CI]		
**Maternal grouping**				
Pregnant womenR	371 (44.70) [41.35-48.10]	61 (85.92) [75.62-93.03]	Ref 0.15 (0.08-0.30)	0.001*
Non-pregnant mothers of under-5	459 (55.30) [51.90-58.65]	10 (14.08) [6.97-24.38]		
**Marital status**				
Never marriedR	22 (2.65) [1.76-3.98]	4 (5.63) [1.56-13.80]	Ref	
Married	781 (94.10) [92.28-95.51]	61 (85.92) [75.62-93.03]	1.17 (0.28-4.94)	0.836
Separated/widowed	27 (3.25) [2.25-4.69]	6 (8.45) [3.16-17.49]	2.40 (0.91-6.33)	0.076
**Parity**				
NoneR	124 (14.94) [12.68-17.53]	23 (32.39) [21.76-44.55]	Ref	
< 3 Children	442 (53.25) [49.85-56.62]	34 (47.89) [35.88-60.08]	0.82 (0.39-1.74)	0.820
^3^3 Children	264 (31.81) [28.73-35.05]	14 (19.72) [11.22-30.86]	0.94 (0.48-1.84)	0.859

* Statistically significant (p<0.05) R:reference

**Socio-demographic and clinical predictors of uptake of malaria intervention:** this study assessed the sociodemographic and clinical predictors for the uptake of malaria intervention by pregnant women and mothers using bivariate and multivariate logistics regression. From the bivariate logistics regression model (Annex 2) significant associations for the uptake of malaria intervention was found for the following; maternal grouping, marital status of the women in the study and parity (i.e. number of children these women in the study have given birth to). Women who were not pregnant at the time of enrollment into the study but already have children less than five years old had an increased significant odds (OR: 7.55, 95% CI: 3.81, 14.93) for uptake of malaria intervention compared to pregnant women (Annex 2). Similar increased likelihood for uptake of malaria intervention was observed for women who have less than three children (OR: 3.50, 95% CI: 1.74, 7.03) and women who have 3 or more children (OR: 1.45, 95% CI: 0.76, 2.75) compared to women who have never given birth (Annex 2). However, the association for uptake of malaria intervention by women who have 3 or more children was not significant; those having less than 3 children showed significant associations for uptake of malaria intervention. In contrast, women who were either widowed or separated from their spouses showed significant reduced likelihood (OR: 0.35, 95% CI: 0.14, 0.88) for uptake of malaria intervention compared to those who were married and those who have never been married (Annex 2). When adjusted for possible confounders, multivariate logistics regression model showed significantly lower odds (OR: 0.15, 95% CI: 0.08, 0.30) for the uptake of malaria intervention by the non-pregnant mothers of children less than 5 years old when compared to the women pregnant at the time of enrollment into the study (Table 2).

**Socio-demographic and clinical predictors of uptake of malaria intervention:** this study assessed the sociodemographic and clinical predictors for the uptake of malaria intervention by pregnant women and mothers using bivariate and multivariate logistics regression. From the bivariate logistics regression model (Annex 2) significant associations for the uptake of malaria intervention was found for the following; maternal grouping, marital status of the women in the study and parity (i.e. number of children these women in the study have given birth to). Women who were not pregnant at the time of enrollment into the study but already have children less than five years old had an increased significant odds (OR: 7.55, 95% CI: 3.81, 14.93) for uptake of malaria intervention compared to pregnant women (Annex 2). Similar increased likelihood for uptake of malaria intervention was observed for women who have less than three children (OR: 3.50, 95% CI: 1.74, 7.03) and women who have 3 or more children (OR: 1.45, 95% CI: 0.76, 2.75) compared to women who have never given birth (Annex 2). However, the association for uptake of malaria intervention by women who have 3 or more children was not significant; those having less than 3 children showed significant associations for uptake of malaria intervention. In contrast, women who were either widowed or separated from their spouses showed significant reduced likelihood (OR: 0.35, 95% CI: 0.14, 0.88) for uptake of malaria intervention compared to those who were married and those who have never been married (Annex 2). When adjusted for possible confounders, multivariate logistics regression model showed significantly lower odds (OR: 0.15, 95% CI: 0.08, 0.30) for the uptake of malaria intervention by the non-pregnant mothers of children less than 5 years old when compared to the women pregnant at the time of enrollment into the study (Table 2).

**Sociodemographic and clinical predictors of the uptake of malaria intervention by SES:** we further assessed the socio-demographic and clinical predictors for uptake of malaria intervention by SES, and the bivariate logistic regression analysis showed a statistically significant association for age in low SES, maternal grouping, and parity for middle SES. Women aged 35 years and above were less likely to utilize malaria intervention (OR=0.08; 95% CI: 0.01-0.46; p=0.005), compared to those 34 years old and below (Annex 3). The non-pregnant mothers of children under 5 years old showed significant higher odds for utilizing malaria intervention (OR=7.62; 95% CI: 3.70-15.71; p=0.001). Pregnant mothers in the middle SES, and women that have one or two children were 3.51 times more likely to utilize malaria intervention (OR=0.13; 95%: 0.06-0.27; p=0.001) compared to women with three or more children in the middle SES. No statistically significant association for marital status, education, parity, and malaria preventive methods with malaria intervention was observed in any of the SES as the probability values for these measured variables were greater than 0.05 (p>0.05) as shown in Annex 2.

**Multivariate logistic regression for middle SES with significant socio-demographic and clinical predators (Adjusted OR):** when adjusted for possible confounders, the multivariate logistics regression model showed a significantly lower odds for maternal grouping (AOR: 0.14, 95% CI: 0.06-0.31, p=0.001) (Table 3). The parity that was initially statistically significant in the bivariate model became non-significant in the multivariate model, which is a pointer that parity is a confounding predictor (AOR: 0.83, 95% CI: 0.37-1.84, p=0.641) of malaria intervention utilization in Middle SES (Table 3).

**Table 3:** multivariate logistic regression for middle SES with significant socio-demographic and clinical predators (Adjusted OR)

Variables	Bivariate OR (95 CI)	p-value	Multivariate AOR (95 CI)	p-value
**Maternal grouping**				
Pregnant women^R^	Ref		Ref	
Non pregnant mothers of under-5^R^	0.13 (0.06-0.27)	0.001*	0.14 (0.06-0.31)	0.001*
**Parity**				
None^R^	Ref		Ref	
< 3 Children	3.51 (1.67-7.37)	0.001*	0.83 (0.37-1.84)	0.641
3 or more children	1.28 (0.65-2.51)	0.481	1.12 (0.55-2.27)	0.756

* Statistically significant (p<0.05) R:reference

## Discussion

This study sought to explain the contribution of socio-economic status as a determinant of access to and uptake of malaria interventions in the study area. Majority of the study population were in the middle SES and multiparous (ie has had more than one child) while about a third had tertiary level of education. Maternal grouping (pregnant versus non-pregnant mothers) and being multiparous had statistical significant relationship with uptake of malaria prevention intervention in this study. Of all possible predictors investigated after adjusting for confounding variables, statistically significant socio-economic predictors for uptake of malaria intervention by the mothers were maternal grouping and age. Non-pregnant mothers of children less than 5 years old were shown to be less likely to use malaria intervention compared to the pregnant women. Similarly, mothers in the low SES, who were aged 35 years and above were also shown to have lower odds for uptake of malaria intervention. Similar to our study, we discovered that findings of the role of lower SES as a predictor for the uptake of malaria intervention from our study support some of the empirical evidence presented by these similar studies in Africa. The recent systematic review on the influence of SES in the burden of malaria in households within sub-Saharan Africa reports that increased risk of malaria infection and the associated burden was significantly influenced by low SES [11]. In Ghana, a study by Nyarko *et al*. [22] revealed that malaria cases were more prevalent among children less than five years old from poor households than those from rich households. Findings from studies conducted among women (both pregnant and non-pregnant) in Madagascar, [23] Senegal, [24] and Nigeria, [25,26] correlates with the present study which showed that lower SES play a significant role in the prevalence of malaria cases in the household and uptake of malaria interventions.

Furthermore, this present study found that non-pregnant women despite being also mothers of children aged less than five years old were less likely to access and utilize malaria interventions compared to the women who were pregnant in the study. This finding is consistent with evidence from studies conducted in sub-Saharan Africa and other malaria-endemic countries which report that despite ongoing malaria prevention and elimination efforts, the rate of utilization of preventive measures is still unsatisfactory especially in sub-Saharan Africa [27,28]. The last demographic and health survey conducted in Nigeria where this study was conducted also had similar findings [26,29]. Possible explanation for improved utilisation of malaria interventions by pregnant mothers compared to their non-pregnant women could be caused by antenatal care (ANC) visits by the pregnant women where they are reminded to use available malaria preventive and curative interventions. Evidence to support this explanation has been demonstrated by Amankwah *et al*. [30] who showed that optimal uptake malaria intervention increased because of ANC visit.

**Limitation:** even though the unique findings from this study as earlier discussed, one limitation worth mentioning is the possibility of recall and information bias because data collected was based on the participant's responses. However, this was addressed during data analysis through exploring several SES predictors for uptake of malaria intervention and adjusting for possible confounders. Another limitation is the study design applied in this study, it is well known that cross-sectional study designs do not establish causality. Thus, we recommend for future studies to use cohort or randomized controlled trials to investigate causal associations of identified SES predictors for uptake of malaria uptake of malaria intervention. Lastly, this study was conducted in a specific city in Nigeria´s south-west region - Ibadan, and we suggest that future studies consider conducting multi center nationwide studies as this would provide well representative and detailed insights that could influence appropriate socio-economic strategies that would improve uptake of malaria interventions to be implemented.

## Conclusion

Despite aforementioned limitations, this study has demonstrated the relationship between socio-economic status of the study population in relation to the uptake of malaria interventions. The results indicate that empowering women in communities is essential to improved health seeking behavior by members of the home and uptake of malaria intervention. The study also provides insights to help health policymakers and stakeholders to refine malaria interventions that would take into perspective the SES of individuals at the grassroots, particularly women. Initiatives are needed to increase awareness of current malaria interventions in communities other than health facilities where pregnant women are more informed due to their ANC visits. However, towards the reduction and elimination of the disease, it is imperative to conduct a holistic study that will include other essential factors like climate and the environment. Studies have shown that there is a relationship between malaria, the environment and climate [8,9]. Therefore, further studies that incorporate environmental factors such as elevation and vegetation including climatic variables are recommended.

### What is known about this topic

- *Socio-economic status (SES) is an important determinant of health seeking behaviour*;
- *To ensure that child health outcome and the well-being of the entire household are improved, women's SES is critical*.

### What this study adds

- *The SES components like age, a maternal category such as currently pregnant or non-pregnant mothers of children under five years of age, and parity (the number of live births) have a substantial influence on the uptake of current malaria intervention measures*;
- *The parity plays a significant role in the uptake of current malaria intervention measures, which should be investigated further in future research; this is because non-pregnant women who were mothers of children under the age of five were found to be less likely to obtain health services, and when the role of the number of live births by these mothers (parity) was investigated further, parity was found to be a confounder, prompting our recommendation for future research*.

## Annexe

**Annex 1**: socio-economic predictor of uptake of malaria intervention

**Annex 2**: socio-demographic and clinical predictors of uptake of malaria intervention using bivariate logistic regression (Unadjusted OR)

**Annex 3**: socio-demographic and clinical predators of uptake of malaria intervention by SES using bivariate logistic regression (Unadjusted OR)
